# Do the anatomical and physiological properties of a muscle determine its adaptive response to different loading protocols?

**DOI:** 10.14814/phy2.14427

**Published:** 2020-04-27

**Authors:** Brad J. Schoenfeld, Andrew D. Vigotsky, Jozo Grgic, Cody Haun, Bret Contreras, Kenneth Delcastillo, Aston Francis, Gilda Cote, Andrew Alto

**Affiliations:** ^1^ Department of Health Sciences CUNY Lehman College Bronx NY USA; ^2^ Departments of Biomedical Engineering and Statistics Northwestern University Evanston IL USA; ^3^ Institute for Health and Sport (IHES) Victoria University Melbourne VIC Australia; ^4^ Department of Exercise Science LaGrange College LaGrange GA USA; ^5^ Sport Performance Research Institute AUT University Auckland New Zealand

**Keywords:** gastrocnemius, high‐load, low‐load, soleus, strength training

## Abstract

It has been proposed that superior muscle hypertrophy may be obtained by training muscles predominant in type I fibers with lighter loads and those predominant in type II fibers with heavier loads.

**Purpose:**

To evaluate longitudinal changes in muscle strength and hypertrophy of the soleus (a predominantly slow‐twitch muscle) and gastrocnemius (muscle with a similar composition of slow and fast‐twitch fibers) when subjected to light (20–30 repetition maximum) and heavy (6–10 repetition maximum) load plantarflexion exercise.

**Methods:**

The study employed a within‐subject design whereby 26 untrained young men had their lower limbs randomized to perform plantarflexion with a low‐load (LIGHT) and a high‐load (HEAVY) for 8 weeks. Muscle thickness was estimated via B‐mode ultrasound and maximal strength was determined by isometric dynamometry.

**Results:**

Results showed that changes in muscle thickness were similar for the soleus and the gastrocnemius regardless of the magnitude of load used in training. Furthermore, each of the calf muscles demonstrated robust hypertrophy, with the lateral gastrocnemius showing greater gains compared to the medial gastrocnemius and soleus. Both HEAVY and LIGHT training programs elicited similar hypertrophic increases in the triceps surae. Finally, isometric strength increases were similar between loading conditions.

**Conclusions:**

The triceps surae muscles respond robustly to regimented exercise and measures of muscle hypertrophy and isometric strength appear independent of muscle fiber type composition. Moreover, the study provides further evidence that low‐load training is a viable strategy to increase hypertrophy in different human muscles, with hypertrophic increases similar to that observed using heavy loads.

## Graphical abstract

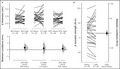

## 1 INTRODUCTION

Human skeletal muscle is composed of two primary types of fibers: type I and type II. Broadly speaking, type I fibers possess endurance‐oriented properties, with slow times to peak tension and a high‐capacity to resist fatigue (Talbot & Maves, 2016). Conversely, type II fibers can be characterized as power‐oriented; they can achieve greater rates of peak tension and shortening velocity but fatigue more rapidly than their type I counterparts (Talbot & Maves, 2016). A majority of the body's muscles are of mixed fiber type consisting of an approximately equal proportion of type I and type II fibers. However, several postural muscles are predominantly type I, which facilitates their ability to sustain repeated muscular contractions over time.

Differences between fiber type compositions are evident in the individual muscles of the human triceps surae. The soleus is composed almost entirely of type I fibers (~80%) whereas the gastrocnemius has a similar composition of both fiber types (Elder, Bradbury, & Roberts, 1982; Gollnick, Sjodin, Karlsson, Jansson, & Saltin, 1974; Johnson, Polgar, Weightman, & Appleton, 1973). This is consistent with the functional role of the respective muscles. The primary role of the soleus is to help sustain posture in the standing position; alternatively, the gastrocnemius has more of a phasic role in carrying out explosive movements at the ankle joint (Vandervoort & McComas, 1983).

Research suggests that resistance training (RT)‐induced hypertrophy is greater in type II fibers compared with type I fibers (Fry, 2004). Fiber composition also appears to play a role in muscular performance, whereby individuals possessing a greater percentage of type I fibers are able to perform more repetitions at 70% of one‐repetition maximum (1RM) compared to those with a higher type II fiber percentage (Douris et al., 2006). Accordingly, it has been proposed that superior muscular adaptations may be obtained by training muscles predominant in type I fibers with lighter loads and those predominant in type II fibers with heavier loads (Fisher, Steele, Bruce‐Low, & Smith, 2011). Indeed, there is speculation that the greater hypertrophic potential of type II fibers generally reported in the literature may be a function of the comparative studies employing high intensities of load, and that low‐load training may be more effective in targeting the endurance‐oriented properties of type I fibers to stimulate further growth (Ogborn & Schoenfeld, 2014). In support of this hypothesis, rodent data indicates greater hypertrophy of the predominantly slow‐twitch soleus muscle when training with lighter versus heavier loads (Padilha et al., 2019). Human research implementing biopsy of mixed fiber‐type quadriceps muscles has produced conflicting results on the topic, with some studies showing differential fiber‐type specific hypertrophic effects between loading conditions (Netreba et al., 2013; Vinogradova et al., 2013) and others showing negligible differences (Lim et al., 2019; Morton et al., 2016). Although discrepancies in findings are not entirely clear, a possible explanation might be related to differences in the intensity of effort employed in these studies. Specifically, studies showing differential adaptations between fiber types seemingly did not train to muscular failure (Netreba et al., 2013; Vinogradova et al., 2013) while those showing no differences reportedly did (Lim et al., 2019; Morton et al., 2016). This has relevance given evidence that training with a high level of effort is necessary to maximize the hypertrophic response of low‐load RT (Burd et al., 2012; Lasevicius et al., 2019). To date, however, the hypothesis has not been tested empirically at the whole muscle level in human muscles predominant in a given fiber type.

The primary purpose of this study was to evaluate longitudinal changes in muscle strength and hypertrophy of the calf muscles between light (20–30 RM–LIGHT) and heavy (6–10 RM–HEAVY) RT routines consisting of plantarflexion exercise. We hypothesized that: (a) strength changes would be greater in the limb performing HEAVY RT; and, (b) hypertrophy would show differential effects in the soleus and gastrocnemius based on magnitude of load, with LIGHT RT promoting greater hypertrophy in the soleus and HEAVY RT promoting greater hypertrophy in the gastrocnemii. A secondary aim was to compare hypertrophic adaptations of the individual calf muscles to determine if differences exist between the responses of muscle with a mixed‐fiber composition (gastrocnemius) versus a predominantly slow‐twitch composition (soleus).

## 2 METHODS

### 2.1 Participants

Participants were 30 healthy male volunteers (height: 175.7 cm; weight: 77.3 kg; body fat: 20.5%; age: 22.5 years) recruited from a university population. This sample size was justified by a priori Monte Carlo simulated precision analysis based on the following assumptions, which are consistent with baseline measures, effects, and relationships observed in the literature (Chow et al., 2000; Schoenfeld, Peterson, Ogborn, Contreras, & Sonmez, 2015) and what we considered to be practically meaningful: (a) soleus would hypertrophy 10 ± 10% from baseline in the HEAVY RT condition; (b) medial gastrocnemius (MG) and lateral gastrocnemius (LG) would hypertrophy 10 ± 10% from baseline in the LIGHT RT condition; (c) muscles on either limb have the same expected value with a correlation *r* = .9; (d) soleus would hypertrophy 15 ± 30% *more* in the LIGHT RT than the HEAVY RT condition, and; (e) MG and LG would hypertrophy 15 ± 30% *more* in the HEAVY RT conditions than the LIGHT RT conditions. The precision analysis showed that 30 participants were sufficient to obtain a 90% compatibility interval (CI) for both effects of interest of ± 0.1 *z*‐score units. To be included in the study, participants were required to meet the following inclusion criteria: (a) be between the ages of 18–35; (b) have no existing musculoskeletal disorders, neuromuscular disorders, lower extremity pain, or prior traumatic injury to the triceps surae/Achilles complex; (c) be free from consumption of anabolic steroids or any other legal or illegal agents known to increase muscle size for the previous year; (d) had not performed regimented RT for the lower body in the past 6 months; and (e) were not currently in a formal athletic program (e.g., varsity athletics, martial arts, etc.).

The study employed an individually randomized within‐group design where each participant performed both LIGHT (20–30 RM) and HEAVY (6–10 RM) RT for the calf muscles. One leg was randomly assigned to the LIGHT condition and the contralateral leg performed the HEAVY condition throughout the study period. A within‐participant design allows for increased precision of effect estimation, especially with the high pre‐post measurement correlations that are observed in studies assessing muscle thickness (MT) (Dankel, Kang, Abe, & Loenneke, 2019). Moreover, the gastrocnemii and solei were studied due to the different distributions of fiber types within each muscle, whereby the soleus is almost entirely a slow‐twitch muscle (Elder et al., 1982; Gollnick et al., 1974; Johnson et al., 1973) while the gastrocnemius displays a mixed composition of slow‐ and fast‐twitch fibers (Gollnick et al., 1974; Green et al., 1981; Johnson et al., 1973). Randomization as to which limb received which stimulus was carried out using block randomization, with two participants per block, in R software (R Core Team, 2019). Ethical approval for the study was obtained from the university Institutional Review Board. Informed consent was obtained from all participants prior to beginning the study. All training and data collection were performed at the same site (academic setting). The methods for this study were preregistered prior to recruitment (https://osf.io/puvxhhttps://osf.io/).

### 2.2 Resistance training procedures

To ensure stimulation of the entire triceps surae musculature (Arampatzis et al., 2006) the RT protocol consisted of performing the seated and standing calf raise exercises for 2 weekly sessions on non‐consecutive days. Training times within each participant were consistent across the duration of the study, but varied between participants to allow for the study to fit within each participant's schedule. A 1‐week familiarization period was provided prior to the study whereby participants performed these exercises unilaterally over 3 non‐consecutive days using their bodyweight for 3 sets of 5, 10, and 15 repetitions per set on Days 1, 2, and 3, respectively. This hypothetically promoted a repeated bout effect and thus helped to prevent unwanted muscle soreness from interfering with training (Clarkson & Sayers, 1999). Prior to training, all participants underwent RM testing for their 8RM and 25RM to determine individual initial training loads (for HEAVY and LIGHT, respectively) in the respective randomized legs for each exercise. RM testing was consistent with recognized guidelines as established by the National Strength and Conditioning Association (Baechle & Earle, 2008).

**FIGURE 1:**
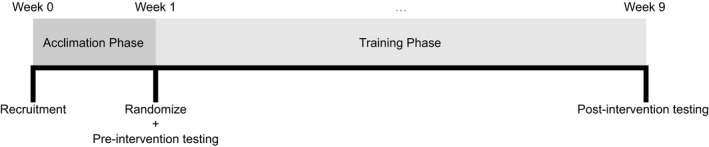
Study timeline. Upon entering the study, all participants went through a 1‐week acclimation phase. After the acclimation phase, participants were randomized and preintervention testing was performed. This order served to prevent potential changes due to acclimation from confounding the results, which are affected by preintervention assessments. The training period lasted a total of 8 weeks, after which, postintervention testing was performed

After the strength testing, participants engaged in 8 weeks of intensive training of the plantar flexors, during which the two interventions were provided concurrently. To minimize any potential confounding effects from exercise order, possible exercise combinations were dispersed throughout the training protocol for each participant, such that every participant started with each exercise‐load combination (e.g., seated LIGHT, seated HEAVY, standing LIGHT, or standing HEAVY) four times throughout the study. Participants performed both LIGHT and HEAVY on the first exercise before moving on to the second exercise, for which participants followed the same order of loading as the first exercise (see Table 1). Participants performed four sets per exercise per session with 90 s of rest afforded between sets and ~3 min of rest afforded between exercises. Sets were carried out to the point of momentary concentric muscular failure—the inability to perform another concentric repetition while maintaining proper form. The load was adjusted for each exercise as needed on successive sets to ensure that participants achieved failure in the target repetition range. Cadence of repetitions was carried out with a controlled concentric contraction and an approximately 2‐s eccentric contraction as monitored by the research staff. All routines were directly supervised by research assistants to ensure the proper performance of the respective routines. Attempts were made to progressively increase the loads lifted each week within the confines of maintaining the target repetition range for each condition. Participants were instructed to refrain from performing any additional resistance‐type lower body training for the duration of the study. A timeline of the study can be found in Figure 1.

**TABLE 1:** Training protocol*

Session 1		Session 2		Session 3		Session 4	
Exercise	Load	Exercise	Load	Exercise	Load	Exercise	Load
Straight‐leg calf raise	Light	Bent‐leg calf raise	Heavy	Bent‐leg calf raise	Light	Straight‐leg calf raise	Heavy
Straight‐leg calf raise	Heavy	Bent‐leg calf raise	Light	Bent‐leg calf raise	Heavy	Straight‐leg calf raise	Light
Bent‐leg calf raise	Light	Straight‐leg calf raise	Heavy	Straight‐leg calf raise	Light	Bent‐leg calf raise	Heavy
Bent‐leg calf raise	Heavy	Straight‐leg calf raise	Light	Straight‐leg calf raise	Heavy	Bent‐leg calf raise	Light

* The protocol was repeated four times for a total of 16 sessions.

### 2.3 Dietary adherence

To avoid potential dietary confounding of results, participants were advised to maintain their customary nutritional regimen and to avoid taking any supplements other than that provided in the course of the study. Dietary adherence was assessed by self‐reported food records using MyFitnessPal.com (http://www.myfitnesspal.com), which were collected twice during the study: 1 week before the first training session (i.e., baseline, during the acclimation phase) and during the final week of the training protocol. Participants were instructed on how to properly record all food items and their respective portion sizes consumed for the designated period of interest. Each item of food was individually entered into the program, and the program provided relevant data as to total energy consumption, as well as amount of energy derived from proteins, fats, and carbohydrates for each time period. To help ensure that dietary protein needs were met, participants consumed a supplement on training days containing 24 g protein and 1 g carbohydrate (Iso100 Hydrolyzed Whey Protein Isolate, Dymatize Nutrition) under the supervision of the research staff.

### 2.4 Measurements

#### 2.4.1 Anthropometry

Participants were told to refrain from eating for 12 hr prior to testing, eliminate alcohol consumption for 24 hr, abstain from strenuous exercise for 24 hr, and void immediately before the test. Participants’ height was measured to the nearest 0.1 cm using a stadiometer; weight was assessed to the nearest 0.1 kg on a calibrated scale, which also provided an estimate of body fat percentage (InBody 770; Biospace Co. Ltd.).

#### 2.4.2 Muscle thickness

Ultrasound imaging was used to obtain measurements of MT of the MG, LG, and soleus. A trained ultrasonographer performed all testing using a B‐mode ultrasound imaging unit (Sonoscape E1). The technician applied a water‐soluble transmission gel (Aquasonic 100 Ultrasound Transmission gel, Parker Laboratories Inc.) to each measurement site, and a 10 MHz ultrasound probe was placed perpendicular to the tissue interface without depressing the skin. Measurements were taken on the posterior surface of both legs at 25% of the lower leg length (the distance from the articular cleft between the femur and tibia condyles to the lateral malleolus). When the quality of the image was deemed to be satisfactory, the technician saved the image to a hard drive and obtained MT dimensions. Images for the MG and LG were measured as the distance from the superficial to deep aponeuroses that borders the soleus. The soleus was measured from the upper and lower aponeuroses separating the muscle. In an effort to ensure that swelling in the muscles from training did not obscure results, images were obtained ≥48 hr after the acclimation phase, as well as after the final training session. This is consistent with research showing that acute increases in MT return to baseline within 48 hr following a RT session (Ogasawara, Thiebaud, Loenneke, Loftin, & Abe, 2012) and that muscle damage is minimal after repeated exposure to the same exercise stimulus over this period of time (Damas et al., 2016). To further ensure accuracy of measurements, three images were obtained for each site and averaged to obtain a final value. The intraclass correlation coefficients (ICCs) from our lab for the MG, LG, and soleus are 0.990, 0.993, and 0.990, and the coefficients of variation are 3.1%, 3.3%, and 3.0%, respectively.

### 2.5 Maximal strength assessments

#### 2.5.1 Muscle strength

To test isometric ankle plantar flexion strength, each participant was secured in a dynamometer (Biodex Isokinetic Dynamometer System 4 Pro) with his hips positioned to 85° flexion, knees in full extension (0°), and testing ankle to 90° (i.e., foot 90° relative to the tibia). Each trial consisted of a maximum voluntary isometric effort that lasted for 5 s, and was followed by 30 s of rest. A total of four trials were performed. To optimize performance, participants were verbally encouraged throughout each trial and were allowed to view the screen for biofeedback. The highest peak net joint moment from the 4 trials was used for analysis. The ICC from our lab for the isometric plantarflexion strength test is 0.76, with a coefficient of variation of 9%. We also endeavored to carry out isometric strength testing of the plantarflexors at 90° knee flexion (i.e., to “isolate” the soleus), but test‐retest reliability was deemed unsatisfactory for this outcome.

### 2.6 Blinding

To minimize the potential for bias, we incorporated two levels of blinding into the design and analysis of this study. First, the principal investigator, who obtained the measurements of the primary outcome, was blinded to group allocation; second, the statistician performed blinded analyses in the form of cell scrambling (MacCoun & Perlmutter, 2015). This was accomplished by having the statistician generate group assignments (i.e., participant ID to coded group ID) and communicating those coded groups directly to one of the research assistants, who then assigned true groups (i.e., left‐heavy or right‐heavy) to the coded labels (0 or 1). The research assistant returned three spreadsheets back to the statistician for analysis; one spreadsheet had the correct label data coupling and the other two contained randomly permuted labels. Only after the analyses were complete did the research assistant unveil which dataset was the correct one.

### 2.7 Statistical analyses

To assess the differential effects of LIGHT versus HEAVY, all data were analyzed in R (version 3.6.1), in which hierarchical linear models were constructed (Bates, Mächler, Bolker, & Walker, 2015). A single model was constructed to obtain two effects, which followed the form:

$$\begin{array}{c} \text{Level}\;1\\ \begin{array}{cc} {\text{post}}_{\mathrm{ij}} & =\beta_{0i}+\beta_{1j}\left(pre_{\mathrm{ij}}\right)+\beta_{2j}\left(intervention_{\mathrm{ij}}\right)+\beta_{3j}\left(pre_{\mathrm{ij}}\times MG_{\mathrm{ij}}\right)\\ & \;+\beta_{4j}\left(intervention_{\mathrm{ij}}\times MG_{\mathrm{ij}}\right)+\beta_{5j}\left(MG_{\mathrm{ij}}\right)+\beta_{6j}\left(pre_{\mathrm{ij}}\times LG_{\mathrm{ij}}\right)\\ & \;+\beta_{7j}\left(intervention_{\mathrm{ij}}\times LG_{\mathrm{ij}}\right)+\beta_{8j}\left(LG_{\mathrm{ij}}\right)+\varepsilon_{\mathrm{ij}} \end{array}\\ \text{Level}\;2\\ \beta_{0j}=\gamma_{00}+r_{0j}\\ \beta_{[i=1-8]j}=\gamma_{i0}, \end{array}$$

where level 1 is hypertrophy (within‐participant), level 2 is between‐participant, and *β_4j_* and *β_7j_* are the effects of interest. These are estimates of the differential effect of the intervention between soleus versus MG and soleus versus LG, respectively. This was estimated for both outcomes in the same model, as soleus was the reference muscle (intercept). Residuals were visually inspected for homoscedasticity.

Secondary analyses were carried out to assess within‐muscle hypertrophy (e.g., the difference in soleus hypertrophy between HEAVY and LIGHT) and strength adaptations. For each analysis, postintervention score was the dependent variable, intervention (i.e., LIGHT or HEAVY) was the independent variable, preintervention scores was a covariate, and there were varied intercepts for each participant so that all analyses were within‐participant, such that the hierarchical linear model took the following form:

$$\begin{array}{c} \text{Level}\;1\\ {\text{post}}_{\mathrm{ij}}=\beta_{0i}+\beta_{1j}\left(pre_{\mathrm{ij}}\right)+\beta_{2j}\left(intervention_{\mathrm{ij}}\right)+\varepsilon_{\mathrm{ij}}\\ \text{Level}\;2\\ \beta_{0j}=\gamma_{00}+r_{0j}\\ \beta_{1j}=\gamma_{10}\\ \beta_{2j}=\gamma_{20} \end{array}$$

where level 1 is strength or hypertrophy (within‐participant), level 2 is between‐participant, and *β_2j_* is the effect of interest.

For all analyses, the bootstrap with 500 replicates was used to obtain bias‐corrected and accelerated 90% compatibility intervals (CI) of the point estimate of each effect. We analyzed data per‐protocol rather than intention‐to‐treat since our interest was in the effect of the intervention rather than its prescription. Finally, to avoid dichotomous interpretations of the results, we did not employ null hypothesis significance testing. Instead, we sought to understand the magnitude of each effect and the range of effects that are compatible with our data, whether they be close to zero or otherwise; that is, an estimation approach (Gardner & Altman, 1986), in line with previous work from our group (Schoenfeld et al., 2019). Thus, rather than interpreting effects from a single test, or set of tests, the results were interpreted on a continuum using all statistical outcomes, in combination with theory and practical considerations (Gardner & Altman, 1986; McShane, Gal, Gelman, Robert, & Tackett, 2019). All other analyses, including sensitivity analyses (e.g., leave‐one‐out), were considered exploratory.

## 3 RESULTS

A total of 26 subjects completed the study protocol; 4 subjects dropped out for the following reasons: personal motives (*n* = 2), non‐compliance (*n* = 1), and injury not related to the training protocol (*n* = 1). Despite the dropouts, we note our precision sample size analysis was conservative; that is, for a 90% CI that encompasses ± 0.1 SDs. Due to these dropouts and perhaps optimistic assumptions, our resulting CIs for our primary outcomes were slightly wider, but not enough to yield practical differences in interpretation. Thus, the sample size was sufficient to draw relevant inferences on the studied outcomes. All subjects completing the study participated in at least > 85% of sessions, with an average attendance of 95%.

### 3.1 Differential growth in soleus versus gastrocnemii

**FIGURE 2:**
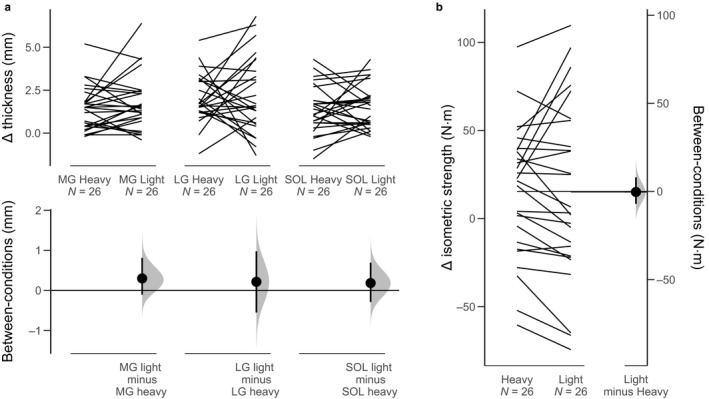
Muscular outcomes. Effect of heavy and light loads on triceps surae muscle growth and plantar flexion strength. (a) Medial gastrocnemius (MG), lateral gastrocnemius (LG), and soleus (SOL) growth within (top) and between (bottom) heavy conditions. (b) Isometric strength outcomes within (left) and between (right) heavy and light conditions. Distributions are bias‐corrected and accelerated bootstrap distributions, and error bars are 90% CIs. All scores are unadjusted (as compared to our statistical models)

To address the primary research question, differential growth in the soleus versus gastrocnemii was assessed. Because the constructs are different–that is, MT in different muscles is not necessarily comparable—all values were *z*‐scored relative to preintervention thicknesses. All of the observed effects were negligible in magnitude. Specifically, MG difference in growth between LIGHT and HEAVY was 0.03 SDs greater than the soleus (Figure 2a). The 90% CI was compatible with *z*‐scores between −0.17 and 0.24. Similar findings were observed in the LG, which exhibited a difference between LIGHT and HEAVY that was 0.02 SDs greater than the soleus (Figure 2a). The 90% CI was compatible with *z*‐scores between −0.24 and 0.28. Leave‐one‐out sensitivity analyses did not reveal remarkable outliers, with *z*‐scores ranging from −0.01 to 0.08 for MG and −0.05 to 0.10 for LG.

### 3.2 Effect of condition on within‐muscle growth

Muscle growth between the LIGHT and HEAVY conditions was generally negligible (see Table 2). Specifically, soleus growth was greater by 0.2 mm in the LIGHT condition, and the data were compatible with values ranging from 0.3 mm in favor of HEAVY to 0.7 mm in favor of LIGHT (Figure 2b). Similar results were obtained for both gastrocnemii: MG growth was greater by 0.2 mm in the LIGHT condition, and the data were compatible with values ranging from −0.2 mm in favor of HEAVY to 0.8 mm in favor of LIGHT; and LG growth was greater by 0.2 mm in the LIGHT condition, and the data were compatible with values ranging from −0.5 mm in favor of HEAVY to 0.8 mm in favor of LIGHT (Figure 2b).

**TABLE 2:** Effect of condition on within‐muscle growth and isometric strength

	Heavy			Light			Between‐condition
	Pre	Post	Change	Pre	Post	Change	
Soleus (mm)	18.8 ± 4.4	20.1 ± 4.6	1.3 ± 1.4	18.2 ± 4.3	19.7 ± 4.6	1.5 ± 1.3	0.2 (−0.3, 0.7)
Medial gastrocnemius (mm)	18.3 ± 3.2	19.7 ± 3.1	1.5 ± 1.3	17.7 ± 3.0	19.5 ± 3.0	1.8 ± 1.6	0.2 (−0.2, 0.8)
Lateral gastrocnemius (mm)	15.9 ± 2.6	17.9 ± 2.5	2.1 ± 1.4	15.6 ± 2.8	17.9 ± 3.2	2.3 ± 2.2	0.2 (−0.5, 0.8)
Isometric plantar flexion (*N*⋅m)	154 ± 48	170 ± 41	15 ± 37	153 ± 47	168 ± 41	15 ± 50	−1.2 (−7.4, 4.5)

### 3.3 Effect of condition on isometric strength

Similar to the changes in muscle growth, negligible differences in strength changes were observed between LIGHT and HEAVY, with a 1.2 N⋅m difference favoring HEAVY (see Table 2). The 90% CI was compatible with values ranging from 7.4 N⋅m in favor of HEAVY to 4.5 N⋅m in favor of LIGHT (Figure 2c).

### 3.4 Between‐muscle growth, irrespective of condition

We explored muscle growth, irrespective of condition, in *z‐*score units. Soleus was found to grow by 0.33 SDs, with 90% CI compatible with *z‐*scores ranging from 0.25 to 0.44. MG grew by 0.44 SDs, with a 90% CI compatible with *z*‐scores ranging from 0.30 to 0.65. LG grew the most, by 0.63 SDs, with a 90% CI compatible with *z‐*scores ranging from 0.52 to 1.00.

## 4 DISCUSSION

This is the first study to directly investigate whether a benefit exists to training muscles based on their fiber type. The study produced several novel and notable findings. First and foremost, muscular adaptations were similar for the soleus (a predominantly slow‐twitch muscle) and the gastrocnemius (a muscle with a mixed composition of both major fiber types) regardless of the magnitude of load used in training. Second, each of the calf muscles demonstrated robust hypertrophy, with the LG showing greater gains compared to the MG and soleus. Third, both heavy and light loads elicited similar hypertrophic increases in the triceps surae. Finally, isometric strength increases were similar between loading conditions.

It has been proposed that muscles composed of primarily slow‐twitch fibers may achieve greater hypertrophy from light‐load training, whereas muscles composed of primarily fast‐twitch fibers may hypertrophy to a greater extent from heavy‐load training (Fisher et al., 2011). Results of the present study do not necessarily support this hypothesis, as neither LIGHT RT nor HEAVY RT differentially influenced hypertrophy in the slow‐twitch soleus and mixed fiber type gastrocnemius muscles, respectively. Our results are in contrast to those of Fujiwara et al. (2011), who reported a greater increase in MT of the soleus compared to the gastrocnemius (12.7% vs. 6.6%) following performance of one daily set of very low load standing plantarflexion exercise (100 repetitions) in a cohort of older women across a 2‐month study period. Discrepancies between the studies may be attributable to several factors. For one, subjects in Fujiwara et al. (2011) performed an unsupervised, home‐based training program, and thus may not have exerted sufficient effort during exercise performance to elicit a robust hypertrophic response in the more fast‐twitch dominant gastrocnemii; alternatively, our study employed fully supervised training with subjects pushed to the point of momentary muscle failure on each set. Moreover, Fujiwara et al. (2011) employed a very high repetition range using only bodyweight as resistance whereas the repetition range in our study, although generally considered high by RT standards, equated to ~¼ the number of repetitions performed in the former protocol. Differences in the populations studied (older women vs. younger men) may also have been a factor, as the aging process results in a gradual loss of fast‐twitch fibers whereby whole muscle develops a slower phenotype with an impaired capacity to generate force (Waters, Baumgartner, Garry, & Vellas, 2010). Such age‐related changes may have predisposed a greater reliance on the soleus in the study by Fujiwara et al. (2011) given the elderly status of participants. Other potential explanations as to the inconsistent findings between studies include differences in training volume and range of motion. It should be noted that Fujiwara et al. (2011) did not compare adaptations to a heavier load protocol, and therefore no conclusions can be drawn as to whether the magnitude of load itself was responsible for the observed results.

Acute research indicates that the soleus muscle has a diminished hypertrophic potential, seemingly due to its predominantly slow‐twitch composition. Data collected in rodents shows that the soleus displays blunted anabolic signaling compared to the fast‐twitch dominant tibialis anterior when subjected to high‐frequency electrical stimulation (Nader & Esser, 2001). Moreover, acute human data demonstrate that the fractional rate of muscle protein synthesis in the soleus following the performance of nine sets of plantarflexion exercise is ~ 200% lower than that commonly reported in exercise protocols involving the knee extensor muscles (Trappe, Raue, & Tesch, 2004). Longitudinal data in rodents generally show that hypertrophy of the fast‐twitch plantaris exceeds that of the soleus following synergist ablation (Chale‐Rush, Morris, Kendall, Brooks, & Fielding, 2009; Roberts et al., 2019); longitudinal human data on the topic when performing traditional RT protocols is lacking. Here, we show that the soleus displayed a mean increase in MT of 7.8%, indicating a robust response to regimented RT. Indeed, changes in MT were similar between the soleus and the mixed‐fiber MG (7.8% and 8.9%, respectively), suggesting that fiber‐type may not play a role in a muscle's hypertrophic potential pursuant to traditional RT. Conversely, the LG displayed greater hypertrophy (13.7%) than both the soleus and MG, clouding the ability to draw strong conclusions on the topic. The discrepancies between findings may be attributed to the inherent contractile properties of the respective muscles, as the LG has been found to have significantly faster twitches compared to the other calf muscles (Vandervoort & McComas, 1983), which conceivably could predispose the LG to greater growth from intense RT given evidence that fibers associated the highest threshold motor units have the greatest hypertrophic potential (Fry, 2004). Moreover, the LG is relatively inactive during standing balance and gait compared to the MG (Duysens, Wezel, Prokop, & Berger, 1996; Heroux, Dakin, Luu, Inglis, & Blouin, 2014), raising the possibility that it has a greater potential training capacity due to underuse. These hypotheses warrant further investigation.

A recent meta‐analysis found that muscle hypertrophy is similar between high‐load (>60% 1RM) and low‐load (≤60% 1RM) training protocols (Schoenfeld, Grgic, Ogborn, & Krieger, 2017). However, the results of this meta‐analysis were limited to the thigh and upper body musculature. Our study adds to the body of literature demonstrating that findings extend to the muscles of the lower leg as well, with similar increases in MT observed for all three triceps surae muscles irrespective of the magnitude of load used in training (9.2% vs. 10.7% for HEAVY and LIGHT, respectively). Collectively, the emerging evidence indicates that hypertrophy can be achieved across a wide spectrum of loading ranges, and it appears that this paradigm holds true for the major muscles of the body regardless of their fiber type composition.

It is generally accepted that strength increases with dynamic RT are greater in programs employing heavier versus lighter loads. However, these findings are specific to dynamic tests of maximal strength such as the 1RM (Schoenfeld et al., 2017); there is a large discrepancy between isometric and dynamic strength changes following training interventions (Jones, Rutherford, & Parker, 1989). The present study found similar changes in isometric strength between conditions, amounting to increases of ~16% to 18% in torque. These results are generally consistent with meta‐analytic data (Schoenfeld et al., 2017) showing a relatively trivial benefit to higher loading on strength (effect size of 0.16) when testing on an isometric instrument. When taken into account with the body of literature, our findings indicate that the transfer of loading magnitude to strength outcomes is dependent on the type of test employed. Specifically, a greater transfer is observed for heavier loads when testing is similar to the conditions used in training (e.g., high‐load dynamic RT has a greater transfer on dynamic, as compared to isometric strength tests).

Our study has several limitations and delimitations that must be considered when attempting to draw practical inferences. First, the results are specific to the triceps surae and cannot necessarily be generalized to other muscle groups. Second, the results are specific to untrained, young men and cannot necessarily be generalized to women, adolescents, the elderly, or those with consistent RT experience. Third, we did not perform biopsies on the muscles of interest, and thus cannot rule out the possibility that individual differences in fiber type between the triceps surae muscles may have influenced results. However, given the compelling evidence that the soleus is a predominantly slow‐twitch muscle and that the gastrocnemii are of mixed fiber types (Vandervoort & McComas, 1983), our findings can be taken with a high degree of confidence. Fourth, MT was measured at a single site; it is conceivable that hypertrophy may have manifested in a non‐uniform manner, which would not have been detected by the methods employed. Fifth, our findings are limited to early phases of RT in untrained individuals; it is possible that hypertrophic potential of different fiber types are reduced with long‐term training programs. Finally, it is conceivable that a cross‐education effect influenced strength adaptations given the within‐subject design, although support for such an effect in the literature is limited to an untrained contralateral limb as opposed to when both limbs perform regimented RT.

## 5 CONCLUSION

Our findings cast doubt on the claim that training muscles based on their fiber composition provides an additional benefit for enhancing muscle strength or hypertrophy. The results also indicate that the triceps surae muscles respond robustly to regimented exercise, and the associated adaptations are independent of load used in the training program provided that sets are performed with a high level of effort. The predominantly slow‐twitch soleus muscle achieved similar increases in MT as the muscle with a mixed fiber type composition (i.e., MG); however, hypertrophy in the LG was superior to the other muscles studied. Finally, we present further evidence that LIGHT RT is a viable strategy to enhance isometric muscle strength and muscle size, as increases in both outcomes were similar between LIGHT and HEAVY RT. Future research employing the muscle biopsy technique for formal muscle fiber‐typing and a similar study design can help to provide clarity on the effects of different load magnitudes on muscles with different fiber‐type populations.

## CONFLICT OF INTEREST

The authors report no conflicts of interest in conducting this study.

## AUTHOR CONTRIBUTIONS

BJS conceived of and designed the study; ADV designed and carried out statistical analysis; AA, GC, KD, AF, and BJS were involved in acquisition of data; all authors were meaningfully involved in interpreting data, and drafting and critically revising the manuscript for intellectually important content.
